# Coupling histone homeostasis to centromere integrity via the ubiquitin-proteasome system

**DOI:** 10.1186/1747-1028-5-18

**Published:** 2010-07-07

**Authors:** Yuko Takayama, Takashi Toda

**Affiliations:** 1 Division of Cell Biology, Institute of Life Science, Kurume University, 1-1 Hyakunen-kohen, Kurume, Fukuoka 839-0864, Japan; 2 Laboratory of Cell Regulation, London Research Institute, Cancer Research UK, 44 Lincoln's Inn Fields, London WC2A 3PX, UK

## Abstract

In many eukaryotes, histone gene expression is regulated in a cell cycle-dependent manner, with a spike pattern at S phase. In fission yeast the GATA-type transcription factor Ams2 is required for transcriptional activation of all the core histone genes during S phase and Ams2 protein levels per se show concomitant periodic patterns. We have recently unveiled the molecular mechanisms underlying Ams2 fluctuation during the cell cycle. We have found that Ams2 stability varies during the cell cycle, and that the ubiquitin-proteasome pathway is responsible for Ams2 instability. Intriguingly, Ams2 proteolysis requires Hsk1-a Cdc7 homologue in fission yeast generally called Dbf4-dependent protein kinase (DDK)-and the SCF ubiquitin ligase containing the substrate receptor Pof3 F-box protein. Here, we discuss why histone synthesis has to occur only during S phase. Our results indicate that excess synthesis of core histones outside S phase results in deleterious effects on cell survival. In particular, functions of the centromere, in which the centromere-specific H3 variant CENP-A usually form centromeric nucleosomes, are greatly compromised. This defect is, at least in part, ascribable to abnormal incorporation of canonical histone H3 into these nucleosomes. Finally, we address the significance and potential implications of our work from an evolutionary point of view.

## Introduction

The timely and selective proteolysis of proteins is essential for cell cycle control. Particularly, ubiquitin-proteasome pathway plays a pivotal role in cell cycle transition and progression [1]. Substrate proteins are ubiquitylated by the enzymatic cascade consisting of ubiquitin-activating enzyme (E1), ubiquitin-conjugating enzyme (E2), and ubiquitin ligase (E3) [2]. The E3 ligase determines the substrate specificity of the pathway. These ubiquitin transferase reactions result in the formation of polyubiquitin chains on substrates, which are recognised by the proteasome, followed by rapid irreversible degradation.

Nucleosomes comprise the repeated units of chromosomal DNAs wrapped around histone octarmers that consist of two sets of each of H2A/H2B and H3/H4 dimer. As DNA replication proceeds, new nucleosomes are formed. Thus, the timing of histone synthesis and DNA replication is coupled, by which newly synthesised histones are rapidly deposited onto replicated DNA [3]. In yeast, previous reports showed that increased histone levels lead to chromosome instability [4] and enhanced DNA-damage sensitivity [5]. Chromosomal instability has been recognised as a hallmark of human cancer [6,7]. However, how the cellular amount of histones is regulated is largely unknown at the molecular levels.

Proper chromosome segregation requires a physical connection between spindle microtubules and centromeric DNAs and this attachment occurs via the kinetochore. CENP-A is a centromere-specific histone H3 variant that is essential for kinetochore formation. CENP-A represents the most likely candidate for the epigenetic mark responsible for maintenance of centromere identity [8,9]. Several recent studies have identified proteins specifically involved in CENP-A loading and centromeric nucleosome formation [10-16]. Interestingly, the cell cycle regulated GATA-type transcription factor, Ams2 in *Schizosaccharomyces pombe *that is required for activation of S-phase specific core histone transcription [17], also promotes the centromeric localisation of CENP-A [18,19]. Ams2 protein levels accumulate at G1-S phase, which is regulated by the ubiquitin-proteasome pathway [20,21]. In this commentary we describe our recent work on the molecular mechanisms of how Ams2 levels are regulated throughout the cell cycle and the deleterious consequences when this intricate system goes wrong.

## Discussion

### Ams2 ensures a cell cycle-specific transcriptional spike of core histone gene expression

**Figure 1:**
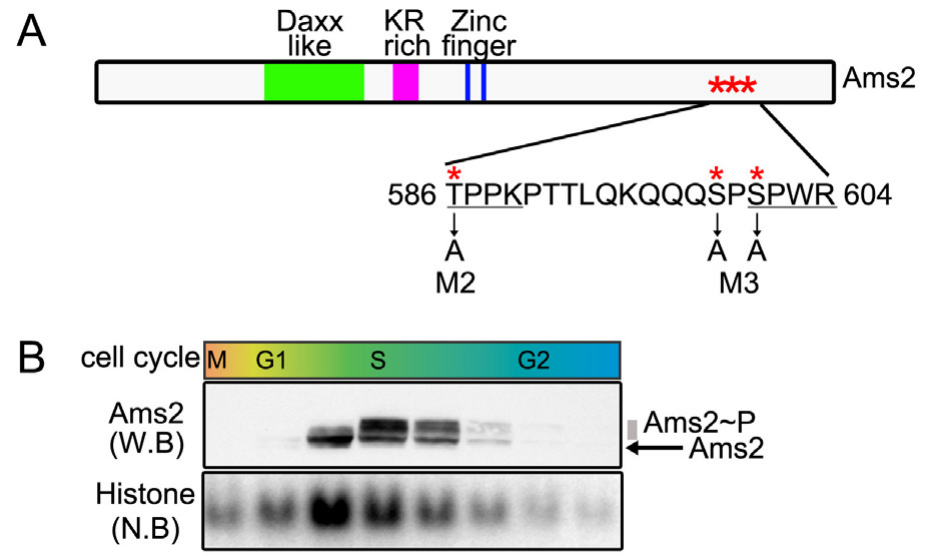
**Ams2 is a cell cycle-regulated GATA type transcription factor**. (A) Schematic structure of Ams2. Structural domains homologous to Daxx (green), amino acid stretches rich in arginine and lysine (magenta) and zinc finger motif (blue) are depicted. Amino acid residues surrounding the phosphorylation sites (asterisks) (which are mutated to alanine in M2 and M3 mutants) and CDK phosphorylation consensus motifs (underlined) are also shown. (B) Fluctuation of Ams2 protein levels during the cell cycle. Wild-type cells were synchronised by centrifugal elutriation. The protein or RNA samples collected every 15 min were assayed by western (anti-Ams2, Ams2 W.B) or northern blotting (histone H4, Histone N.B).

Ams2 was originally identified as one of the multicopy suppressors of the temperature sensitive (ts) *cnp1-1 *mutant [19], defective in the centromere-specific histone H3 variant CENP-A [22]. Interestingly genomic sequences encompassing canonical histone H4 genes were also isolated as other multicopy suppressors from the same screening. This raised the possibility that Ams2 could be involved in transcriptional control of histone genes. Indeed Ams2 is a member of the GATA factors containing Daxx and zinc finger DNA binding motifs (Figure 1A).

Subsequent analysis explicitly showed that Ams2 is a major, if not the sole, transcription factor required for the S-phase specific transcriptional spike of all of the core histone genes [17]. Ams2 directly binds a consensus "AACCCT-box" that exists in the 5' franking regions of these histone genes. Note that *cnp1*^+ ^is not regulated by Ams2 nor does it contain the "AACCCT-box" in its promoter region. A genome-wide search for the "AACCCT" motif followed by binding experiments suggested that core histone genes would be the major, if not the sole, targets of Ams2 (note that only one gene, SPAC631.02 encoding a conserved bromodomain protein, contains the "AACCCT-box" in its 5' flanking region, to which Ams2 binds [17]).

In the absence of Ams2, transcriptional activation of histone genes does not occur during S phase, leading to very low levels of histone transcription throughout the cell cycle [17]. Cells with deletion of *ams2*^+ ^manage to divide, but exhibit a number of defective phenotypes, including slower growth and frequent chromosome missegregation [19].

### Ams2 protein shows cell cycle-dependent oscillation due to varied protein stability

In parallel with a spike of histone gene expression, Ams2 protein accumulates in the nucleus at S phase [17,19]. In sharp contrast, little, if any of this protein is detected during mid G2 and M phase (see Figure 1B). That is, Ams2 protein appears to disappear from cells upon completion of S phase or at the beginning of G2 phase. *ams2*^+ ^transcription is upregulated from G1 to S phase, which is consistent with Ams2 protein accumulation during this period. However, this does not account for the rapid disappearance of Ams2 protein after S phase. Instead, this suggests the sudden destabilisation of Ams2 in G2 phase. Indeed the Ams2 protein is unstable and rapidly degraded at G2 and M phase (half-life ~20 min), but is markedly stabilised in S phase (half-life > 60 min). In G1 cells, the Ams2 is degraded, although at a much slower rate (half-life ~40 min). In summary, the Ams2 protein is unstable during G2 and M phase, partially stabilised in G1, and stable in S phase. Changes in protein stability coupled with transcriptional regulation, therefore, account for oscillations of Ams2 levels during the cell cycle.

### Ams2 protein is degraded via the SCF^Pof3^-proteasome pathway

Regulation of protein stability through the ubiquitin-proteasome pathway is a key mechanism underlying various cellular processes [1]. To address the involvement of this pathway in Ams2 stability, we examined the half-life of this protein in proteasome mutants. As suspected, Ams2 degradation during G2 phase was substantially suppressed, which was accompanied by massive accumulation of ubiquitylated Ams2.

There are two ubiquitin ligases that regulate cell cycle progression, Anaphase Promoting Complex/Cyclosome (APC/C) and Skp1-Cdc53/Cullin-1-F-box (SCF) [23,24]. As described earlier, Ams2 protein is rapidly degraded in G2 phase. We posited that APC/C is unlikely to be involved, as this ubiquitin ligase should be inactive at G2 phase, when Ams2 is degraded. This raised the possibility that the SCF is responsible. Subsequent analysis showed that this is in fact the case.

SCF consists of various subcomplexes, each of which contains different F-box proteins, components responsible for binding specific substrates. Our previous work and that from other laboratories showed that fission yeast contains 18 F-box proteins [25-27] (see Table 1). Three of these F-box proteins, Pop1, Pop2 and Pof3, play crucial roles in cell cycle progression and coordination [28-34]. Half-life analysis using individual mutants showed that the Ams2 protein was stabilised in the *pof3 *deletion mutant, but not in *pop1 *or *pop2 *mutants. Furthermore, Pof3 did bound to Ams2, substantiating the suggestion that SCF^Pof3 ^is an E3 ligase responsible for degradation of Ams2.

**Table 1:** Fission yeast F-box proteins

			homologue		SCF/	
F-box protein	motifs	recognition protein	yeast	human	*non-SCF	References
Pop1	WD40	Cig2, Cdc18, Rum1	Cdc4	Fbw7	SCF	[30,34,46]
Pop2	WD40	Cig2, Cdc18, Rum1	Cdc4	Fbw7	SCF	[30,34,46]
Pof1	WD40	Zip1	Met30	βTrCP	SCF	[47]
Pof2	LRR		Grr1	?	SCF	[25]
Pof3	LRR, TPR	Mcl1, Ams2	Dia2	?	SCF	[25,28,48]
Pof4	none		Ela1	ElonginA	SCF	[25]
Pof5	none		YDR360c	?	SCF	[25]
Pof6	CAAX		Rcy1	?	non-SCF	[49,50]
Pof7	none		Hrt3	?	SCF	[25]
Pof8	none		Ufo1	?	non-SCF	[25,51]
Pof9	none		YBR280c	?	SCF	[51]
Pof10	WD40		YML088w	?	SCF	[52]
Pof11	WD40		?	βTrCP	SCF	[51]?
Pof12	none		?	?	non-SCF	[25,51]
Pof13	none		?	?	non-SCF	[25,51]
Pof14	none	Erg9	?	?	SCF	[26]
Fbh1	DNA helicase	Atf1	?	Fbh1	SCF	[53-58]
SPAPB1A10.14	none		?	?	SCF	[51]

*Assignment of some F-box proteins (Pof6, Pof8, Pof12 and Pof13) as 'non-SCF' is based upon the fact that these F-box proteins, albeit binding Skp1, do not have the conserved proline residue within the F-box motif that is critical for direct interaction between F-box and Cullin-1 [59]. In fact, binding experiments showed this is the case [51]. The only exception is Pof9, which does not contain the proline residue, but it acts as a component of SCF.

### Cdc7 homologue Hsk1 is a kinase responsible for Ams2 degradation

**Figure 2:**
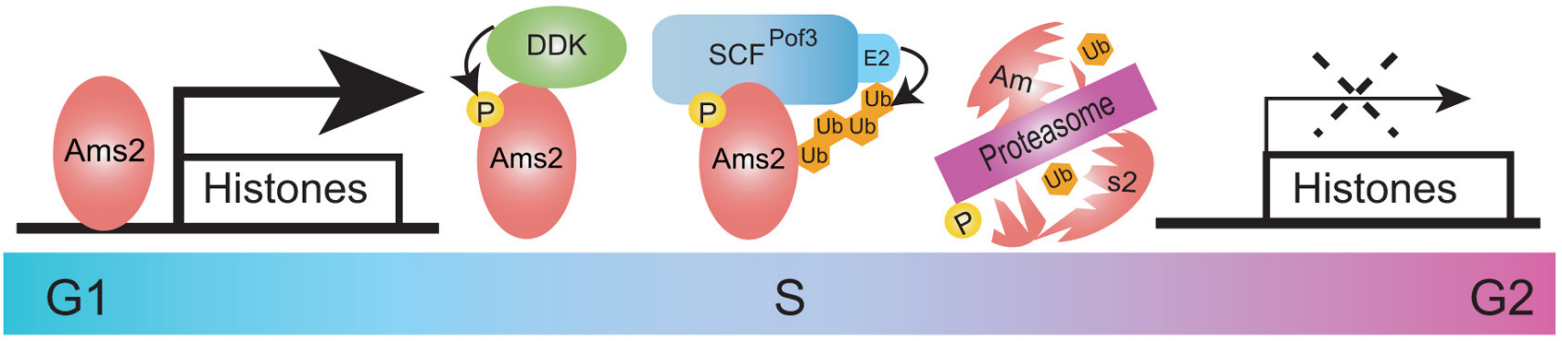
**A model for the mechanism of Ams2 protein oscillation**. Ams2 binds 5' flanking regions of core histone genes, thereby activating histone gene expression. Thereafter, Ams2 is phosphorylated by DDK and its phosphorylated form is recognised by SCF^Pof3^-ubiquitin ligase. Ubiquitylated Ams2 is then degraded by the proteasome, resulting in repression of histone gene transcription.

F-box proteins normally recognise phosphorylated substrates [35]. Indeed, Ams2 protein shows a cell cycle-specific band-shift on gel electrophoresis, in which a faster migrating form appears coincident with a peak of histone gene expression (Figure 1B). This faster form is then replaced by slower phosphorylated forms, followed by abrupt disappearance [17]. Half-life measurements of Ams2 using various *cdc *mutants showed that Cdk1/Cdc2 is not required for Ams2 instability (see below for details). As Ams2 phosphorylation occurs in S-phase, we examined the involvement of the S-phase kinase DDK (Dbf4-dependent protein kinase, Cdc7 in budding yeast and Hsk1 in fission yeast). In the *hsk1 *mutant, Ams2 is hypo-phosphorylated and highly stabilised. This result suggested that Hsk1-DDK is a kinase responsible for S-phase phosphorylation of Ams2, which leads to SCF^Pof3^-mediated proteolysis (see Figure 2 for scheme).

### Potential cooperative roles between CDK and DDK in Ams2 proteolysis

In vitro kinase reaction showed that Ams2 is a substrate of Hsk1. Several subsequent experimentations narrowed down potential phosphorylation sites to three residues (586T, 599S and 601S, Figure 1A, asterisks). Mutagenesis analysis of these sites (M2, M3 and M2&M3 in Figure 1A) indicated that 599S and 601S are phosphorylated by Hsk1-Dfp1 in vitro. Expression of individual mutants in fission yeast, followed by half-life analysis, supported that these threonine and serines are required for Ams2 phosphorylation and subsequent proteolysis.

It is notable that when a phospho mutant, i.e., a stable form of Ams2 (M2&M3), is expressed from its native promoter, its mRNA levels are slightly but reproducibly decreased compared to those of wild-type Ams2. This result indicates that some monitoring system, possibly a feedback mechanism, would operate in maintaining the proper cellular levels of histone and/or Ams2. Further studies are necessary to uncover the identity of this regulatory loop.

Intriguingly, two sites of the above three residues are within the CDK consensus (586TPPK589 and 601SPWR604, Figure 1A, underlined). As described earlier, our stability data, however, suggested that Cdc2/Cdk1 is not required for mobility shift of Ams2 phosphorylation or degradation. How could this apparent discrepancy be explained and what is the relationship between CDK consensus sites and DDK? Here, we posit two scenarios. One is that Hsk1 is capable of phosphorylating CDK consensus sites that are found in Ams2. As consensus phosphorylation sites for DDK remain to be determined, this notion cannot be ruled out. Another possibility, which is more appealing, is that CDK and DDK act in concert for Ams2 phosphorylation. According to this scenario, CDK would phosphorylate T586 and S601, thereby promoting subsequent S599 phosphorylation by Hsk1. Despite this, Cdc2-dependent phosphorylation may not be absolutely essential as far as activation of Hsk1 and phosphorylation of S599 and/or S601. It is tempting to speculate that CDK may play a role in Ams2 phosphorylation as a priming kinase for DDK, although this needs further scrutiny. It is also possible that in addition to ubiquitylation and degradation, individual phosphorylation by CDK and DDK could make distinct contributions toward Ams2 function, such as nuclear localisation and chromatin binding (see below). Further biochemical analysis will be necessary to draw solid conclusions regarding this issue.

### Spatiotemporal control of Ams2 stability during S phase

**Figure 3:**
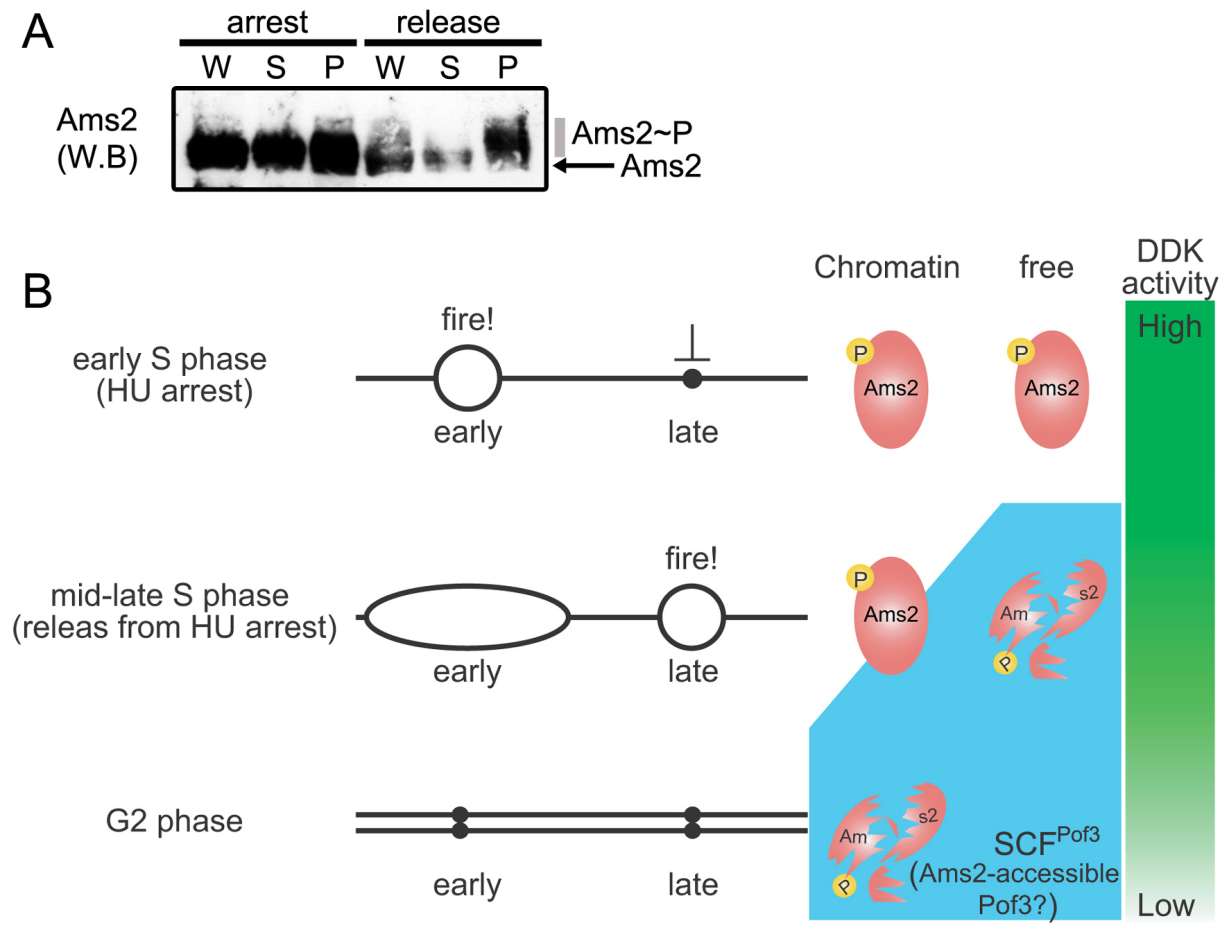
**Molecular behaviour of Ams2 throughout S phase**. (A) Chromatin fractionation assay. Wild-type cells were arrested with HU (arrest) and released into the HU-free fresh medium for 15 min (release). Whole-cell extracts (W) were spun and separated into soluble supernatant (S) and chromatin-bound pellet (P). Western blotting was performed with anti-Ams2 antibody. (B) A speculative model for spatial and temporal regulation of Ams2 during S and G2 phase. Early replication origins are activated in the presence of HU (fire), whereas the firing of late origins is blocked. During this period, DDK has high kinase activity, and thereby Ams2 proteins become phosphorylated, although Ams2 is stable. When cells are released from HU arrest, phosphorylated Ams2 is still detected only on chromatin, in which the chromatin-free phosphorylated fraction may be degraded via SCF^Pof3^. As DNA replication progresses further and completes (G2), SCF^Pof3 ^may be able to interact with chromatin-bound phosphorylated Ams2. (see text for details)

Ams2 proteolysis requires phosphorylation by DDK, which is active during S phase. However, as shown in Figure 3A, Ams2, both soluble and insoluble (chromatin-bound), is stable when cells are arrested at S phase with HU treatment. How can we reconcile this result? Interestingly, once released from HU block, Ams2, which is bound on chromatin, is stable, whereas phospho-Ams2 in the soluble fraction is selectively degraded (Figure 3A and 3B). Perhaps during early S phase or when DNA structure checkpoint is activated (triggered by HU treatment), Pof3 may be prevented from accessing phospho-Ams2. As S phase proceeds further, only soluble Ams2 is capable of interacting with Pof3, but chromatin-bound Ams2 is still not. We postulated that Ams2 stability is regulated in a dual manner, temporally via S-phase progression and spatially via its DNA/chromatin binding activity, which may be dependent on accessibility of SCF^Pof3 ^to Ams2.

### Centromeric nucleosomes are compromised by constitutive histone synthesis

As described earlier, histone synthesis is under strict cell cycle control through Ams2. It would be thus of particular interest to see the physiological effects derived from constitutive Ams2 expression. Intriguingly, several expected and unexpected phenotypic consequences have been observed. It may not be surprising that excess Ams2 is harmful for cell growth with induced chromosome loss. This detrimental effect is exaggerated in the presence of the *hsk1 *mutation, consistent with the fact that this mutant already has increased levels of histones ascribable to Ams2 stabilisation.

It is unexpected that chromatin architectures at the core centromere are dramatically altered. Micrococcal nuclease (MNase) experiments showed that the core centromere region, which normally exhibits non-structured patterns (smears with MNase digestion), was almost indistinguishable from that in other euchromatin regions, namely regular ladder patterns. This indicates that structural compositions of histones in this core centromere region may be altered by Ams2 overproduction. Indeed, in these Ams2-overproducing cells, a substantial amount of H3 histones is incorporated into the centromere. It is important to note that in these cells CENP-A^Cnp1 ^still exists at the core centromere as in wild-type. That is, the number of nucleosomes is increased, in which nucleosomes comprising canonical H3 or CENP-A^Cnp1 ^appear to coexist in the core centromere, leading to formation of more tightly packed nucleosomes in this region. Perhaps this abnormal centromere status would be the reason, at least in part, for growth arrest and massive chromosome loss phenotypes. Analysis of euchromatin (e.g., around the actin gene) in Ams2-overproducing cells also indicates that the overall number of nucleosomes is increased. In summary, induced overexpression of all the core histone genes forces formation of more nucleosomes on both euchromatin and centromere regions.

These results indicate that despite the existence of multiple mechanisms by which to ensure the formation of centromere-specific nucleosomes [10-16], overproduction of a single transcription factor, Ams2, is sufficient to interfere with these regulatory networks. We envisage that this edge of wall status of histone homeostasis is indeed the reason that fission yeast has developed DDK-phosphorylation- and SCF-mediated proteolysis of Ams2 during the cell cycle. It would be interesting to address other defects except at the centromere in Ams2-overexpressing cells.

### Evolutionary conservation of histone homeostasis from yeast to human

Are the mechanisms underlying cell cycle-dependent histone homeostasis conserved? The Ams2 protein, however, seems not to be conserved in other species, which may suggest that what we have found is a fission yeast-specific regulatory system. Nonetheless as described below, we envision that although individual organisms have developed different strategies to maintain a proper level of histones, they implement the universal regulatory system, that is, the ubiquitin-proteasome pathway.

**Figure 4:**
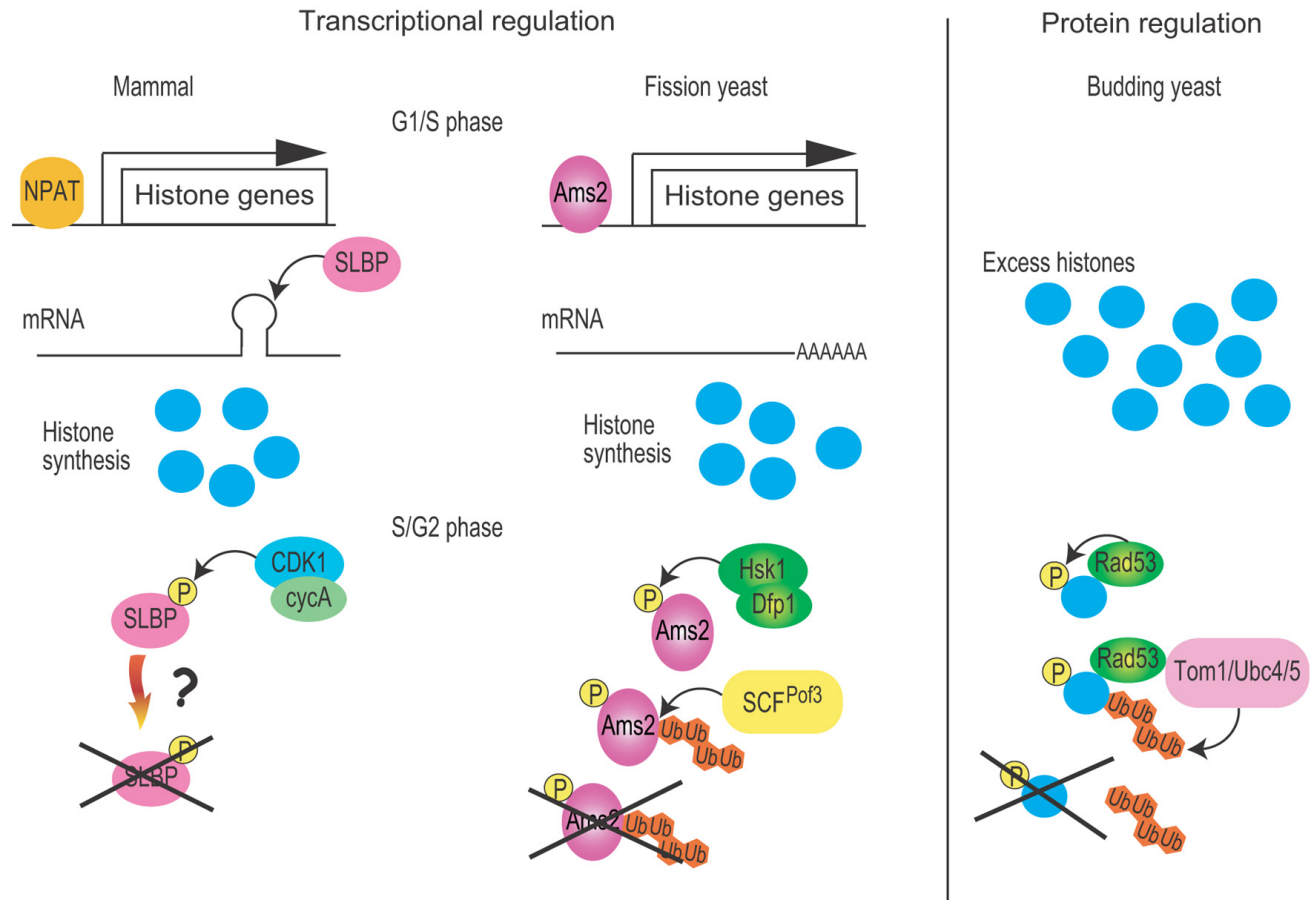
**General view of the species-specific regulation of histone homeostasis**. In mammals (left) [45], histone transcription is activated by NPAT (Nuclear protein, ataxia-telangiectasia locus) and SLBP is then bound to the 3' end of histone mRNA, by which it prevents degradation of mRNAs, resulting in synthesis of histone proteins. At the end of S phase, CDK1-cyclinA (cycA) phosphorylates SLBP to trigger its degradation, restraining further transcription of histone mRNAs. In fission yeast (middle) [20], Ams2 activates histone transcription at G1/S phase. At the S/G2 phase, Ams2 is phosphorylated by DDK, leading to degradation via the SCF^Pof3^-ubiquitin proteasome pathway. In budding yeast (left) [36], excess histones are recognised and phosphorylated by Rad53. The histone-Rad53 complex is recognised by the Ubc4/5 (E2) and Tom1 (E3) and is polyubiquitylated. Histones with a polyubiquitin chain are degraded by the proteasome.

In budding yeast, histone levels, in particular those of non-chromatin forms, are regulated by phosphorylation and the ubiquitin-proteasome pathway [36]. In this regulatory system, excess free histones are phosphorylated by Rad53 (homologues of fission yeast Cds1 and human CHK1/2), resulting in polyubiquitylation via the Ubc4/Ubc5 (E2) and Tom1 (E3) and subsequent degradation by the proteasome (Figure 4, right). Although not shown in fission yeast, it is noteworthy that in budding yeast and fly, CENP-A protein levels are strictly regulated by the ubiquitin-proteasome system [37,38]. Therefore, at least in yeast, the histone levels are under the control of phosphorylation and the ubiquitin-proteasome system.

In mammalian cells, unlike yeast or plants, the 3' ends of histone mRNAs contain a stem-loop structure. This specific structure is recognised by stem-loop binding protein (SLBP), by which mRNA half-life is determined. Interestingly, SLBP is synthesised at S phase and is rapidly degraded at the end of S phase. Furthermore, SLBP degradation is triggered by phosphorylation through CDK-cyclinA (Figure 4, left) [21,39]. However, the molecular basis of SLPB degradation is not known. Given the degradation of fission yeast Ams2 and budding yeast free histones, we envisage that SLBP is degraded via the ubiquitin-proteasome pathway. Hence, although the molecular details of regulatory systems may not be the same among individual species, the underlying principles as to how histone homeostasis is maintain via ubiquitin-mediated proteolysis would be conserved from yeast to human beings.

### Concluding remarks

Through the pioneering work of Hartwell and colleagues [4], it is known that ectopic expression of core histone genes has a deleterious effect on chromosome transmission fidelity. However, the underlying mechanism of this toxicity has remained a mystery for decades. We have shown that in fission yeast the levels of the Ams2 transcription factor, which is responsible for periodic histone gene expression, are regulated via the SCF^Pof3 ^ubiquitin-proteasome pathway and that Ams2 proteolysis plays a critical role in maintaining chromosome stability. Importantly, constitutive synthesis of Ams2, and therefore excess core histones, results in structural alterations of centromeric nucleosomes. Our study, therefore, provides the first clear answer to the above question--this is at least in part ascribable to the impairment of centromere structure and function.

**Figure 5:**
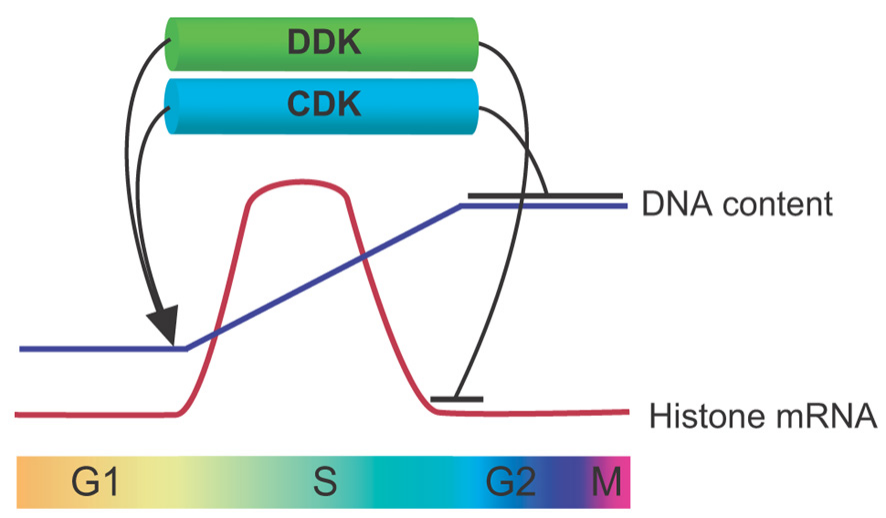
**Cooperative roles of CDK and DDK in cell cycle progression from G1 to G2 phase**. CDK and DDK promote G1-S phase transition and are required for proper S phase progression (arrows). In addition, these two kinases act in concert during G2 phase. CDK is required for prevention of chromosomal DNA re-replication, whereas DDK is essential to repress untimely histone gene expression, thereby helping ensure centromere integrity.

Our work illuminates interesting, unexpected parallels between CDK and DDK during the cell cycle, in particular their roles during G2 phase. It is well established that CDK is important to prevent re-replication of chromosomes upon completion of S phase [40]. In contrast, it is generally believed that DDK's major, if not sole, role lies in S phase, although previous work in yeast indicated that this might not always be true [41,42]. Our study clearly showed that DDK is needed to degrade Ams2 during G2 phase, thereby preventing unnecessary histone gene expression during the post-S phase period. Hence, CDK and DDK not only share roles in S phase initiation and progression, but are also required to secure proper passage of G2 phase upon completion of S phase. CDK is required for restraining deleterious re-replication of the genome, whereas DDK prevents extra histone transcription, which would result in global and local genome disturbance, including centromere malfunction (Figure 5).

Cooperative action between CDK and DDK is consistent with their structural and regulatory framework. Both kinases consist of catalytic and regulatory subunits, in which their regulatory subunits play a decisive role in kinase activities, localisation and substrate recognition. Intriguingly, the levels of cyclins (for CDK) and Dbf4/Dfp1 (for DDK) are under the control of the APC/C ubiquitin ligase [43,44]. Although phylogenetically neither catalytic nor regulatory subunit of CDK and DDK is evolutionarily close, common regulatory mechanisms suggest that CDK and DDK are, at least functionally, much more intimate and cooperative than currently thought.

## List of abbreviations

APC/C: Anaphase Promoting Complex/Cyclosome CDK: cyclin-dependent kinase; DDK: Dbf4-dependent kinase; E1: ubiquitin-activating enzyme; E2: ubiquitin-conjugating enzyme; E3: ubiquitin ligase; HU: hydroxyurea; MNase: Micrococcal nuclease; LRR: leucine rich repeats; NPAT: Nuclear protein; ataxia-telangiectasia locus; SCF: Skp1-Cdc53/Cullin-1-F-box; SLBP: stem-loop binding protein; TPR: tetratricopeptide repeat.

## Competing interests

The authors declare that they have no competing interests.

## Authors' contributions

YT designed and performed experiments and prepared the figures. YT and TT wrote the paper. Both authors have read and approved the final manuscript
